# Application of an adsorptive-thermocatalytic process for BTX removal from polluted air flow

**DOI:** 10.1186/2052-336X-12-89

**Published:** 2014-05-28

**Authors:** Roohollah Rostami, Ahmad Jonidi Jafari

**Affiliations:** 1 Department of Environmental Health Engineering, Semnan University of Medical Sciences, School of Health, Semnan, Iran; 2 Department of Environmental Health Engineering, Iran University of Medical Sciences, School of Health, Tehran, Iran

**Keywords:** Aromatic organics, Air pollution, Chemical process, Nanoparticles, Zeolite

## Abstract

**Background:**

Zero valent iron and copper oxide nanoparticles (30-60 nm) were coated on a bed of natural zeolite (Clinoptilolite) with 1-2 mm grains and arranged as a dual filter in a stainless steel cylindrical reactor (I.D 4.5 cm and L = 30 cm) to investigating the coated bed removal efficiency for BTX. The experiments were conducted in three steps. First, with an air flow of 1.5 L/min and temperature range of 38 (ambient temperature) to 600°C the BTX removal and mineralization was surveyed. Then, in an optimized temperature the effect of flow rate and pollution loading rate were surveyed on BTX removal.

**Results:**

The BTX removal at 300 and 400°C were respectively up to 87.47% and 94.03%. Also in these temperatures respectively 37.21% and 90.42% of BTX mineralization were achieved. In the retention times of 14.1 s and 7.05 s, respectively 96.18% and 78.42% of BTX was removed.

**Conclusions:**

According to the results, this adsorptive-thermocatalytic process with using Clinoptilolite as an adsorbent bed and combined Fe^0^ and Cu_2_O nanoparticles as catalysts can be an efficient and competitive process in the condition of high flow rate and high pollution loading rate with an adequate process temperature of 350°C.

## Background

Volatile organic compounds (VOCs) are one of the indoor and ambient air pollution causes. These compounds, after the suspended particles, have the most frequency and variety of emission. VOCs include a wide range of organic compounds with a boiling point lower than 180°C or vapor pressure greater than 0.01 Kpa in 25°C [1]. Some VOCs are used as solvents that are abundantly used in industrial operations and processes [2]. BTX (Benzene, Toluene and Xylenes) is of the VOCs that are considered as predominant pollutants and have adverse effects on the health and environment [3]. Petroleum derivates contain some concentration of BTX. Detrimental effects of the BTX on the central nervous system, respiratory, genome and excretory system are pointed in literatures [2]. The BTX known to cause of air, soil and ground water pollution around the petroleum and natural gas producing sites, gas stations, and petroleum reservoirs [4,5]. The airflow containing VOCs might be treated by various removal processes like adsorption, biofiltration and oxidation. Zeolites are one of the capable materials for adsorption of VOCs. In the case that the flow contains a high concentration of VOCs, the zeolite is used as a sorbent that can be then cremated or recycled. For the low concentrations of VOCs, the absorption can be combined with an oxidative catalyze process. Advantage of zeolite application rather than carbon bases such as activated carbon is its capability to be used for the flow which contains fewer pollutant concentration and moisture. In these cases, application of the zeolites with a high level of silica which are hydrophobic, are so effective [6].

Thermal VOCs removal processes require relatively high temperatures, so some catalysts are used for conducting the thermal process in lower temperatures. In related studies, according to the type of catalyst and other conditions, different temperatures are obtained for VOCs removal. The nanoparticles are widely used as catalyst in various fields and environmental issues too. For an example of air pollution controlling case, Catalytic incineration of benzene on metal oxide catalysts has been investigated and the results showed a complete destruction of benzene at the temperature of 300°C and 5.5% (weight percent) of copper nanoparticles on TiO_2_. Whit the results, the percent of nanoparticles has affected the benzene destruction. But, When it increased to 7%, the removal efficiency dropped [7]. Complete oxidation of naphthalene on metal oxide catalysts, showed the catalytic properties of metal oxides such as CuO [8]. Some other studies have shown destruction of the volatile organic compounds by some other nanoparticles such as iron nanoparticles [9-11]. Also, removal of VOCs is experimented with different sorbents. Good results of BTEX removal by adsorption on the natural zeolite of Clinoptilolite are achieved in previous studies [12]. In this work we designed the purpose of our study on investigating the elimination of the BTX form polluted air flow by an adsorptive-thermocatalitic process, simultaneous potential for adsorption and catalysis, with application of Cu_2_O and Fe^0^ nanoparticles as catalytic agents on a modified Clinoptilolite bed as absorbent. Also a new configuration of bed arrangement was applied in this work to enhance catalytic fortune of the process for BTX removal.

## Methods

This work was an experimental research and we used crashed natural zeolite grains with 1-2 mm diameter size. 1 and 2 mm pour size sieves were used to separation of the grains with desired size. The zeolite was containing about 85% Clinoptilolite. It was from Semnan province of Iran and provided from Afraznd Inc. Two stages acidic modification were carried out on the sieved zeolite. In each stage the grains poured in a flask containing 1 N hydrochloric acid and shook for six hours at 50°C. After the second stage, it rinsed several times with distilled water and then dried at 180°C for two hours [12].

Copper oxide (Cu_2_O) and zero-valent iron (ZVI) or Fe^0^ nanoparticles with a particles size of 30-60 nm were provided from Plasma Chem Inc [13]. Coating of zeolite grains with the nanoparticles was performed by draining the grains in a dispersed suspension of nanoparticles. Each kind of nanoparticles was poured in an Erlenmeyer flask containing distilled water and sonicated several minutes to make a uniform suspension. Then the zeolite grains added to the flasks and shook moderately for two hours. Finally, the flasks containments were dried slowly at 80°C for ten hours. The added value of nanoparticles was so that it was 4.5 wt% of zeolite [7,14]. The required nanoparticles to gain 4.5 wt% was determined by preliminary tests.

**Figure 1:**
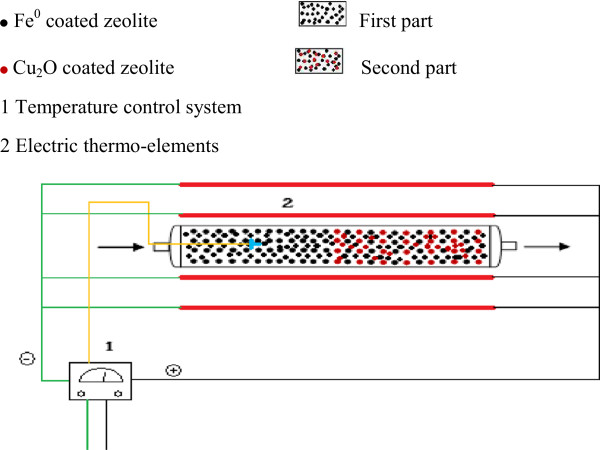
Scheme of the reactor packed with nanoparticle coated zeolite.

The BTX removal experiments were carried out in a continuous system with a packed stainless steel cylinder (I.D = 4.5 cm, length = 30 cm) as the reactor. The reactor's bed was a two parted bed. 200 grams of ZVI nanoparticles coated zeolite placed in head of the reactor for first part. Then 200 grams of 1:1 mixture of ZVI nanoparticles coated zeolite and copper oxide nanoparticles coated zeolite were placed in the reactor as the second part. Figure 1 shows the scheme of the reactor. The results of prior studies were led to this configuration of the bed [11,15,16]. Required temperature provided by six 250 W electric thermo-elements that were arranged around the cylinder. The synthetic polluted air flow was prepared by passing a controlled portion of the inlet flow from a BTX containing flask which is more schematically detailed in the references [15,17]. Removal of BTX compounds with this system was experimented with an inlet flow rate of 1.5 L/min or 56.62 m^3^/m^2^.h, m^2^ is the cross section area of the reactor, at different temperatures. The flow rate gives a space velocity (SV) equal to 188.7 h^-1^ and an empty bed retention time (EBRT) of 19.08 s. The temperature range was from 38°C, the ambient temperature without adjustment, to 600°C. After obtaining the optimum temperature, the experiments were conducted under optimum temperature and variable BTX concentration or pollution loading and then variable flow rate (1 and 2 L/min, 37.75 m^3^/m^2^ h and 75.49 m^3^/m^2^ h). With having these flow rates, SV and EBRT of the reactor respectively were 125.82 h^-1^ and 28.6 s for 1 L/min, similarly 251.6 h^-1^ and 14.31 s for 2 L/min. The pollution load setting was done by controlling the portion of air flow rate through the BTX flask and also the flask temperature.

Sampling flow rate was 100 ml/min and the sample volume was two liters. SKC charcoal tubes were used for BTX sampling by placing it in sampling current line [17,18]. After the sampling, BTX compounds were extracted from the charcoal using 2 ml of Carbon disulfide (CS_2_) in 5 ml vials. The vials were shaken for 10 minutes during the extraction [18]. A gas chromatograph (Chrom Tech) was used as an analytical system. It was equipped with a flame ionization detector (FID). A 25 m silicone capillary column with 0.32 mm of internal diameter was fitted in GC for BTX analysis. The analysis performed according to NIOSH manual of analytical method [18]. One μL of each extracted sample was injected to GC with injection temperature of 180°C and detector temperature of 250°C.

The concentration of CO_2_ was monitored in inflow and outflow during the experiments. GAS-TEC CO_2_ detector tubes were used for CO_2_ examination according to the factory instruction [17]. Blank volume of the reactor and filter net volume was determined by the water saturation test [19,20].

## Results

**Figure 2:**
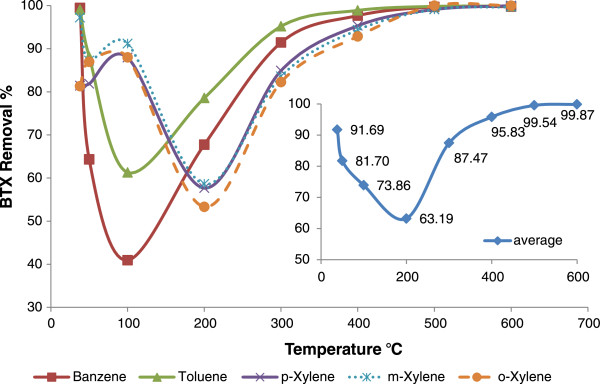
BTX removal in adsorptive-thermocatalytic process as a function of temperature.

The obtained results showed an average BTX removal up to 91% at ambient temperature (38°C). A drop occurred in average of BTX removal efficiency with increasing of temperature. There were some oscillations in the removal of each compound until 200°C and the removal trend of xylenes is not as same as for benzene and toluene. But, in higher than 200°C the removal efficiency for all of the BTX compounds began to rise and difference among the removal of pollutants was reduced. The greatest difference among the removal of the pollutants was occurred at 100°C. These results are eliminated in Figure 2. It shows a jump (63.19% To 87.47%) in BTX removal from 200 to 300°C and in higher than this point, it increased some more smoothly and in higher than 500°C the BTX elimination raised to around 100%. The concentration and loading properties of BTX for this step of the experience are presented in Table 1.

**Table 1:** Inlet BTX concentration and loading rate for the range of temperature (38-600°C)

	**Benzene**	**Toluene**	**p-Xylene**	**m-Xylene**	**o-Xylene**
**Concentration (ppm)**	317.73	61.44	10.30	6.95	4.97
**μg/cm**^**3**^**.h**^**a**^	157.49	30.45	5.10	3.44	2.46
**μg/gB.h**^**b**^	90.08	17.42	2.92	1.97	1.41
**μg/gC.h**^**c**^	2001.68	387.08	64.87	43.77	31.33

^a^Micro gram per each cubic centimeter of bed per hour; ^b^Micro gram per each gram of bed per hour; ^c^May named as weight hourly space velocity (WHSV), micro gram per each gram of catalyst per hour.

**Figure 3:**
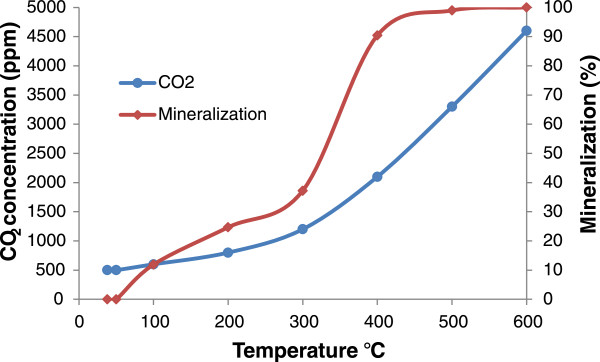
**Outlet CO**_**2 **_**concentration and mineralization of BTX in adsorptive-thermocatalytic process as a function of temperature.**

Concentration of CO_2_ in the inlet was constant and equal to 500 ppm. In the exhaust air flow it was increased with increasing the reactor temperature. This increase began after 50°C and it was gradually raised to 1200 ppm until 300°C. It was increased more sharply at higher than 300°C. Figure 3, shows the effect of temperature on CO_2_ concentration of the reactor outlet. Based on the CO_2_ concentrations and BTX removal information, the BTX mineralization efficiency (conversion to CO_2_) was calculated and is shown in Figure 3. Equation 1 shows balanced mineralization equations for BTX.

$$\left(\mathrm{Benzene}\right)\;2C_{6}H_{6}+15O_{2}\to 12CO_{2}+6H_{2}O$$

$$\left(\mathrm{Toluene}\right)\;C_{7}H_{8}+9O_{2}\to 7CO_{2}+4H_{2}O$$

$$\left(\mathrm{Xylenes}\right)\;2C_{8}H_{10}+21O_{2}\to 16CO_{2}+10H_{2}O$$

Eq. 1 Balanced equations of the BTX mineralization.

The results showed that with increasing the temperature from 300 to 400°C, the BTX mineralization greatly increased from 37.21% to 90.42% and at 600°C almost 100% of the BTX mineralization was obtained.

**Figure 4:**
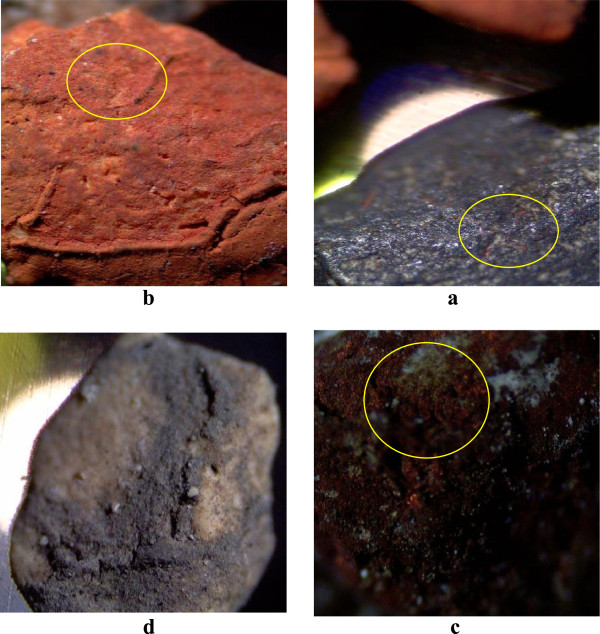
**The coated zeolites befor and after application of high temperature. a**: Zeolite grain with Zero-valent iron nanoparticles coating after using in the reactor less than 400°C, **b**: Zeolite grain with Zero-valent iron nanoparticles coating after using in the reactor at 400°C, **c**: Zeolite grain with Copper oxide (I) nanoparticles coating after using in the reactor less than 400°C, **d**: Zeolite grain with Copper oxide (I) nanoparticles coating after using in the reactor at 400°C.

**Figure 5:**
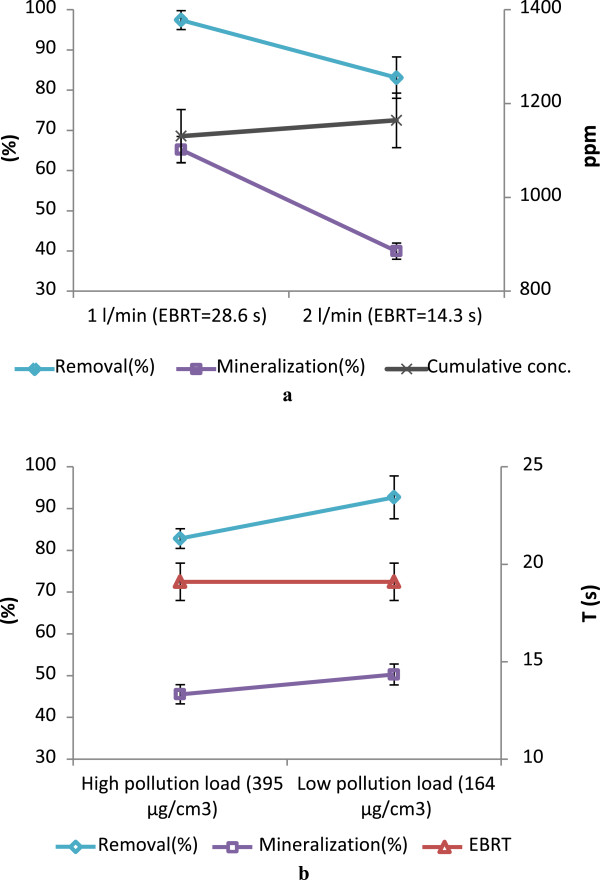
BTX removal in adsorptive-thermocatalytic process with different flow rates (a) and BTX loading rates (b).

Oxidation of the used nanoparticles of ZVI and copper oxide was occurred around 400°C and the oxidation was intensified in higher temperatures. Figure 4 shows the zeolite granules containing ZVI and copper oxide nanoparticles that were used at 400°C and under it. Oxidation of the nanoparticles was not so considerable at around 350°C. So, the experiments followed at 350°C as the reactor temperature with variable pollutants loading rate and polluted air flow rate. Figure 5, shows the BTX removal in the mentioned conditions. According to the results, increasing of the pollutants concentration, caused an increase in the total pollution load and was lead to reduction of BTX removal efficiency. These results are presented in Table 2. In both high and low pollution loads, toluene removal efficiencies with 86.09% and 98.73% were more than of the other pollutants. The lowest removal efficiency allocated to benzene when the pollution load was high. In low pollution load, o-xylene has the lowest removal efficiency. The results showed that the BTX removal at the flow rate of 1 L/min with 97.43% was more than 2 L/min with 83.09%.

**Table 2:** BTX Concentration and removal effisinecy in the adsorptive-thermocatalytic process with different pollution loads and flow rates

	**Pollutant**	**Concentration (ppm)**	**μg/cm**^**3**^**.h**	**μg/gB.h**	**μg/gC.h**	**Removal (%)**
High pollution load	Benzene	1818.92	901.59	13.64	303.15	78.37
	Toluene	486.16	240.99	3.65	81.03	86.09
	p-Xylene	42.01	20.82	0.32	7.00	84.05
	m-Xylene	35.74	17.73	0.27	5.96	82.85
	o-Xylene	7.21	3.57	0.05	1.20	82.75
Low pollution load	Benzene	792.92	393.03	5.95	132.15	92.65
	Toluene	172.07	85.29	1.29	28.68	98.73
	p-Xylene	11.82	5.85	0.09	1.97	93.58
	m-Xylene	9.17	4.56	0.07	1.53	90.01
	o-Xylene	3.51	1.74	0.03	0.59	88.55
1 l/min flow rate	Benzene	910.01	300.72	6.83	151.67	95.64
	Toluene	200.99	66.42	1.51	33.50	98.52
	p-Xylene	5.62	1.86	0.04	0.94	94.32
	m-Xylene	9.61	3.18	0.07	1.60	98.67
	o-Xylene	4.32	1.44	0.03	0.72	100
2 l/min flow rate	Benzene	927.16	612.75	6.95	154.53	76.13
	Toluene	209.14	138.21	1.57	34.86	87.74
	p-Xylene	7.06	4.68	0.05	1.18	82.19
	m-Xylene	11.01	7.29	0.08	1.84	88.53
	o-Xylene	9.86	6.51	0.07	1.64	80.84

The obtained result of saturation test showed that the total volume, blank volume and net volume per 400 grams of the used zeolite in the reactor were respectively 463.68, 234.9, 228.78 mL. With these results and inlet air flow rate, the retention time in the reactor was 14.1 s in flow rate of 1 L/m and 7.05 s for 2 L/min.

Table 3, shows the CO_2_ concentration in outlet of the reactor in different conditions of pollution load and flow rate. In high pollution load, the CO_2_ concentration was higher than the low pollution load and there was further concentration of CO_2_ in the reactor outlet when the flow rate was 1 L/min rather than 2 L/min. according to these results and BTX concentrations, in flow rate of 1 L/min the mineralization of BTX was 65.2% and for the flow rate of 2 L/min it was 39.96%. Also for the high and low pollution loadings, it was respectively 45.53% and 50.27%.

**Table 3:** **Concentration of CO**_**2 **_**in exhaust air of the reactor with different pollution loads and flow rates**

	**CO**_**2 **_**Concentration (ppm)**
**2 l/min flow rate**	2300
**1 l/min flow rate**	4000
**Low pollution load**	2800
**High pollution load**	4800

## Discussion

The results showed that the BTX removal in ambient temperature was more than higher temperatures until 400°C. But the researches have shown that the catalysis process at low temperatures is very weak and removal of pollutants at low temperatures is due to the adsorption phenomena [21,22]. Pollutants desorption occurs with increasing the temperature and although some of the pollutants are decomposed, But most of them again will be released by desorption in the environment [12]. Das et al. found that the favorable temperature for adsorption of toluene on activated carbon fiber was 50°C and at temperature exceeding 100°C, almost no adsorption was occurred in their research [23]. According to these results, BTX removal is reduced with increasing the temperature until 200°C in result of the desorption process. The catalysis was not so effective until this temperature and it was not been able to compensate the loss of BTX removal due to the decreased adsorption. Then a jump in BTX removal occurred after 200°C. It shows that, catalysis process of BTX removal was rapidly progressed. Oxidation and reduction activity of the metallic nanoparticles are declared in other researches and it is shown that greater oxidation of pollutants were occurred in higher temperatures [24-26]. Also the metals are involved in the oxygen activation and producing the free radicals like H and ^•^OH in presence of water vapor that can participate in pollutants' degradation [27,28]. For example the flowing reaction can be occurred about benzene as primary reactions in the oxidation process (Equation 2).

$$O+H_{2}O\to^{•}\mathrm{OH}+\mathrm{OH}$$

$$C_{6}H_{6}{+}^{•}\mathrm{OH}\to C_{6}H_{5}\mathrm{OH}+H$$

$$H+O_{2}\to \mathrm{OH}+O$$

$$C_{6}H_{6}+\mathrm{OH}\to C_{6}H_{5}+H_{2}O$$

$$O+C_{6}H_{6}\to C_{6}H_{5}O+H$$

Eq. 2 Primary oxidation reaction of benzene in the catalytic process.

According to the results, the oxidation of nanoparticles in temperatures above 400°C is being faster and ZVI nanoparticles quickly are converted to hematite (Fe_2_O_3_) with reddish-orange color and copper oxide (Cu_2_O) nanoparticles to CuO with black color. Zou showed that, in a photocatalysis process using TiO_2_–SiO_2_, toluene has been removed by adsorption and catalyzed mechanism. As the toluene, water and CO_2_, which are the products of organic compounds' decomposition, were present in the exhaust air [29]. So, Increased CO_2_ concentration above 200°C indicates the increase of catalytic BTX removal and better mineralization of pollutants in these temperatures. Garetto and Apesteguıa gained the temperature of 312°C for decomposing 50% of Cyclopentanone using a catalyst Pt/Al_2_O_3_[30]. Lu and Wey also recommended the temperature of 250°C using activated carbon with transition metals such as copper, iron, nickel and cobalt for the concurrent removal of VOC and NO [31]. These results are consistent with the results of the present study, because the results show the increase of the catalytic decomposition and mineralization of pollutants above 200°C. Also in the study of Li et al, the complete removal of benzene on Al_2_O_3_ substrate with 29.5% of cobalt oxide was obtained in 400°C. They also showed the relationship between weight percentage of the metal coated on the bed and decomposition of benzene. They achieved the complete removal of benzene using 15.7% of cobalt on a bed of silica at 330°C [32]. Although the percentage of the used metal was more than of nanoparticles in our study with 4.5 wt%, but the results are almost identical with. These results shows that the appropriate temperature for the catalytic removal of the BTX was around 250-400°C. So, the increasing of BTX removal from 200 to 300°C and further mineralization over 300°C in this study is a function of more catalytic activity due to higher temperature.

According to the results there were some differences in the removal of Xylenes with benzene and toluene at the temperatures lower than 200°C. If the adsorption was been the dominant removal process in this range of temperature, It can be due to lower volatility of Xylenes compared to benzene and toluene that the increase in temperature until 200°C could not desorbed them from the bed. Different act and reactivity of xylenes also is reported by Franco et al. They stated that the xylenes faster react with ozone rather than benzene and decomposition of benzene was not completely in higher flow rate, 0.5 L/min, vs 0.2 L/min with a ozone concentration of 35 mg/L. They used a carbon bed in the process. So one can suppose that the higher vapor pressure of benzene, 10 kPa at 20°C, rather that xylenes, 0.8 kPa, cause the benzene rapidly scape form the reactor and decomposition by ozone [3].

According to the results of this research, increasing of the pollution loading was leads to decrease in removal of BTX. This abatement of removal efficiency is obvious influence of the pollution loading rate on the process removal performance. Therefore, we can say that the drop of benzene removal efficiency in the high pollution loading could also be due to its high concentration.

When the air flow through the filter increased from 1 to 2 L/min, the retention time dropped from 14.1 s to 7.05 s with the EBTR equal to 14.31 s. This reduction of the retention time resulted in decrease of the BTX removal. These results indicate that, mineralization of the BTX is more decreased than its removal efficiency by the drop in retention time (see Figure 5a). Faulty mineralization or incomplete decomposition of BTX compounds, for example benzene, may lead to production of the compounds such as Phenyl, Oxy phenyl, Dioxy phenyl and Hydroxy phenyl in the initial stages of decomposition. With the subsequent decompositions, they are converted to other organic compounds and eventually when it completely oxidized turns into CO_2_ and water [33,34]. With these results and results of BTX mineralization in different pollution loading rates (see Figure 5b) it can be stated that, the retention time and flow rate were more effective than the pollution loading, due to higher concentration, on the mineralization of the BTX. According to the results, despite the minor imposed pollution mass on the filter unit volume in the flow rate of 2 L/min rather than the condition with a lower BTX concentration and pollution loading; it has less mineralization of BTX. These results confirm the most impact of retention time versus the pollutant concentration on the BTX mineralization.

Removal of BTX by biofiltration using compost-activated carbon media showed a removal efficiency of ≥90% for each of the BTX compounds with inlet concentrations of ≥200 ppm and a gas loading rate of 17.6 m^3^/m^2^ h [35]. The minimum gas loading rate in our work is 1 L/min, which is equal to 37.75 m^3^/m^2^ h and the BTX concentration except than xylenes was higher than Abumaizar's study. But removal of all of the BTX compounds in adsorptive-thermocatalytic process was higher than 94% and this is higher than biofiltration process's removal efficiency.

Lu and et al. removed the BTX using a trickle-bed air biofilter. In their study, in the flow range of 6.02–8.6 L/m^3^ h, removal efficiencies of each compound were greater than 80% with a loading of 143 g BTX/m^3^ h [36]. Also it is very lower than the removal efficiency of this study with 490.5 g BTX/m^3^ h and flow rate of 393.4 m^3^/m^3^ h in low pollution loading rate condition. These results indicate that, this process can be used for BTX removal in the concentrations and flow rates very higher than for the biofilters.

Sleiman et al. were studied removal of toluene by photocatalytic oxidation with using TiO_2_ as photocatalyst [37]. The inlet concentration in their study was 20–400 ppbv and flow rate was 70–350 mL/min. In their results, the toluene conversion was up to 90–100% with a slight influence of inlet concentration under these conditions, and the mineralization was varied from 55 to 95%. They reported that the flow rate and inlet concentration exhibit a negligible effect on mineralization and has shown to be strongly inhibited by the increase of relative humidity. The inlet concentration is much lower than the inlet concentrations for our study. Also it is same about the flow rate. However, the conversion of toluene in both studies is close together. But, unlike the Sleiman's report, our results showed that the inlet concentration and especially flow rate strongly was influenced the mineralization. It seems that in the Sleiman's study, the flow rate variation was not adequate to reduce the retention time significantly and influence the mineralization. So, in all their flow rates, there was enough time for the process to complete decomposition of toluene. Toluene removal efficiencies up to 78 ± 2% were obtained under optimal conditions in a Heterogeneous photocatalytic process with TiO_2_. These results obtained under the condition that, the toluene inlet concentration was 23–465 ppmv and gas residence time was 17–115 s [38]. Almost the same results obtained in other study [39], and in a adsorptive photocatalytic process with combined nano-scale titania–montmorillonite–silica exhibited Almost 100% of degradation efficiency within 120 min with about 500 ppb initial concentration [40]. Degradation efficiencies of <2% for benzene, 11 ± 2.4% for toluene, 3 ± 1% for ethylbenzene, 1 ± 1% for o-xylene, and 3 ± 0.4% for m and ρ-xylene were obtained in a non-thermal plasma based air purifying system [41]. These results are very lower than ours. But the applied flow rate in the process is 320 L/min. This flow rate is so much than our study, and this may be a reason of the lower removal efficiency. In another study, Au was loaded (1.5 wt.%) on the supports (ZnO, Al_2_O_3_ and MgO) by a colloidal deposition method in a catalytic oxidation of benzene, toluene and *p*-xylene [42]. The results showed the benzene conversion exceeded 80% at 150°C for the Au/ZnO catalyst. Loading rate in the process was 2.1 g/m^3^ for benzene, 0.6 g/m3 for toluene and 0.4 g/m3 for p-xylene. The flow rate in the process was 20 mL/min. The percent of used metal in the process and the temperature is lower than this study. But the flow rate and loading rate are much less compared to this work.

## Conclusions

According to the results and above discussions it can be inferred that; by low temperatures, adsorptive-thermocatalytic process BTX removal acts with adsorption and with increasing of temperature, the BTX adsorption reduces. In the temperatures lower than 300°C, catalytic ability of the process is not enough for properly decomposition of BTX and above 400°C the nanoparticles were rapidly oxidized, but in 350°C the particles oxidation was not significant and decomposition of BTX is reasonable. So the optimum temperature for adsorptive-thermocatalytic process was obtained 350°C.

With decreasing retention time, drop in mineralization of the pollutants is greater than drop in pollution removal. Increasing of the flow rate and decreasing retention time has a greater effect than pollution loading rate on the mineralization of BTX compounds in this adsorptive-thermocatalytic process. This shows the importance of contact time for mineralization of the pollutants in this process.

According to the results, this adsorptive-thermocatalytic process with using Clinoptilolite as an adsorbent bed and combined Fe^0^ and Cu_2_O nanoparticles as catalysts can be an efficient and competitive process in the condition of high flow rate and high pollution loading rate compared to other catalytic process and biofiltration for removal of BTX from polluted air stream.

## Competing interests

The authors declare that they have no competing interest.

## Authors’ contributions

AJJ: conception and design, administration, guide on analysis and interpretation and supervision. RR: conception and design, data collection, analysis and interpretation, statistical expertise and writing the manuscript. Both authors have read and approved the final manuscript.
